# Atypical Presentation of Acute Coronary Syndrome-Not ST Elevation: A Case Report

**DOI:** 10.1155/2012/182379

**Published:** 2012-02-28

**Authors:** Nicola Vitulano, Graziano Riccioni, Antonio Trivisano, Carlo Palumbo, Linda D'aloia, Elena Ricci Barbini, Nino Leone, Miriam Placentino, Silvia Cappetti, Pierluigi Pellegrino, Rocco Perrella, Vincenzo Ferrara, Gaetano Prencipe, Lorenzo Pellegrino, Matteo Melchionda, Matteo Vitulano

**Affiliations:** ^1^Department of Cardiology, Catholic University of Rome, Via dei Gelsi 15, 71043 Manfredonia, Italy; ^2^Cardiology Unit, “San Camillo de Lellis” Hospital, Foggia, Manfredonia, Italy; ^3^Emergency Unit, “San Camillo de Lellis” Hospital, Foggia, Manfredonia, Italy; ^4^Anesthesiology Unit, “San Camillo de Lellis” Hospital, Foggia, Manfredonia, Italy

## Abstract

We describe the unexpected case of a 70-year-old man, with medical history of ischemic heart disease and surgery for aneurysm of abdominal aorta, who comes to the emergency department complaining of *low-back pain* without other symptoms or signs of organic failure. After a few hours we see a deterioration of physical conditions with pulmonary oedema, increase of blood pressure, changing in the ECG pattern, and worsening of left ventricular function with progressive increase of biomarkers for myocardial necrosis. So this pain has revealed the premature symptom of an acute coronary syndrome (ACS). After a short time a subsequent cardiac arrest complicates the clinical situation. After resuscitation, the patient undergoes successfully to coronary angiography and performed a percutaneous transluminal coronary angioplasty (PTCA).

## 1. Case Presentation

**Figure 1:**
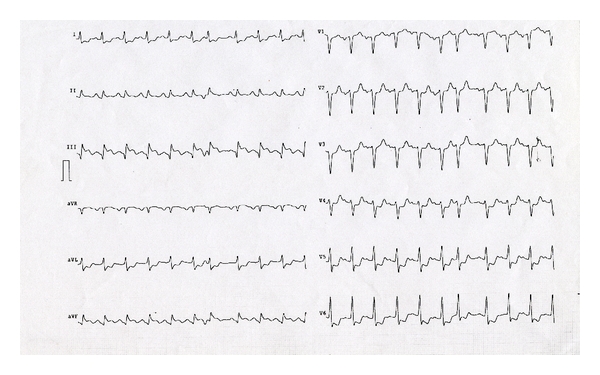
ECG recorded after some hours the onset of the sintomatology, it presents diffuse ST-segment depression to D1 and a VL leads and from V4 to V6 precordial leads with supraventricular ectopic beats.

**Figure 2:**
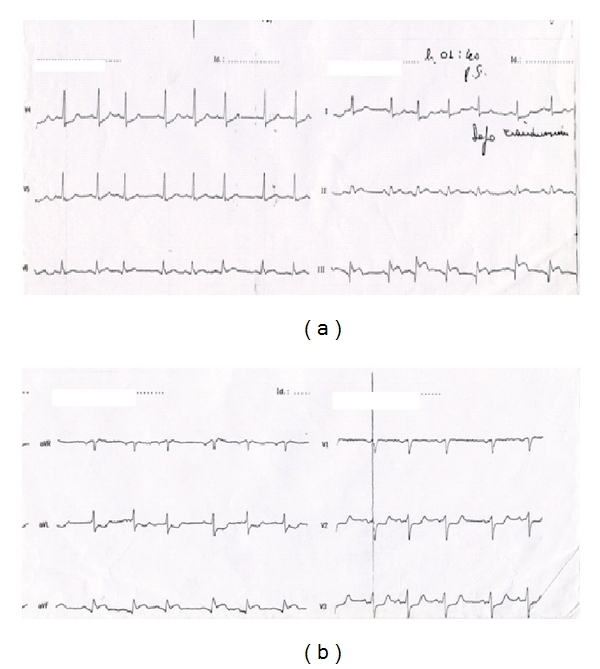
After external cardiac massage and defibrillation we assist to resuscitation with sinus rhythm.

A 70-year-old caucasian man comes to the emergency room for *low-back pain* of recent onset following a strain (to lift a weight). He does not report angina, chest pain, and other symptoms associated. Clinical examination shows good general conditions, rhythmic pulse, presence of a IV tone; nonpathological signs to medical examination of pulmonary field. The value of blood pressure is 100/60 mmHg; isosphygmic the arterial wrists; hypo-sphygmic the pedidies; the tibial back of both side. The additional cardiovascular risk factors are hypertension and diabetes type II. The remote pathological anamnesis of about two years before is characterized of ischemic heart disease (a previous coronary angiography showed critical stenosis of left anterior descending artery, treated with distal PTCA and not critical stenosis of left coronary artery) and surgery for aneurysm of the abdominal aorta. Regarding this medical history and the persistence of this pain with its features to be dull and continue, ECG, computerized tomography (CT) (for chest and abdomen, with contrast means), and monitoring of the indexes of myocardial necrosis are performed. ECG presents sinus rhythm to 92 beats per minute, findings of inferior necrosis, and scarce progression of the R wave in V1–V3 precordial leads comparable with previous ECG (shown by patient). The CT does not show neither signs of aneurysm nor dissection of the aorta. The extemporaneous biomarker monitoring in the department of emergency instead finds value of TnT≃2, 0 *μ*g/L. After a few hours and after the admission into department of cardiology, laboratory examination shows LDH 591 UI/L (range 240–489 UI/L), myoglobine 112,73 ng/mL (range 11,6–73 ng/mL), and troponin I 6,37 ng/mL (range 0,00–0,04 ng/mL). The patient begins to present progressive wheezing and cold sweating, with blood pressure of 150/100 mmHg, rhythmic pulse, and tachycardia, reduced vesicular breathing, rattles to averages and small beads to both the bases, and crackles in the middle pulmonary fields. The ECG presents diffuse ST-segment depression to D1 and a VL leads and from V4 to V6 precordial leads with supraventricular ectopic beats (Figure 1). The echocardiography points out severe and global dysfunction of left ventricle with hypocontractility of the cardiac walls and ejection fraction of 20% and hypokinetic right ventricle (PA 160/105 mHg). After therapy for ACS and a diuresis of 200 cc, the symptomatology is always wheezing with a PA of 130/80 mmHg. During the transfer to intensive care unit for a coronary angiography we see the onset of ventricular flutter with cardiac arrest. After external cardiac massage and defibrillation we assist to resuscitation with sinus rhythm (documented by ECG performed in the emergency room after reanimation) (Figures 2(a) and 2(b)). The patient regains consciousness for the transport to perform coronary angiography that documents multivessel illness with a culprit lesion of right coronary artery treated with stenting, stenosis of 75% of left coronary artery, diffuse atherosclerosis of posterior descending artery, and previous stent without occlusion or intrastent proliferation.

## 2. Discussion

Myocardial infarction (MI) is the rapid development of myocardial necrosis caused by a critical imbalance between oxygen supply and demand of the myocardium. This usually results from plaque rupture with thrombus formation in a coronary vessel, resulting in an acute reduction of blood supply to a portion of the myocardium. Although the clinical presentation of a patient is a key component in the overall evaluation of the patients, often health care providers do not recognize symptoms of MI. The diagnosis of MI is a whole of clinical, biochemical, and electrocardiographic features that range from a typical sintomatology to electrocardiographic features and biomarkers that help overall when this symptoms are not typical such as in this case report. Regarding the development of this clinical case, it is part of what is defined acute coronary syndrome-not ST elevation (ACS-NSTE); traditionally its several clinical presentations have been distinguished such as prolonged anginal pain at rest, new onset severe angina, recent destabilization of previously stable angina, and post-ima angina. The angina typically is a retrosternal pressure or heaviness radiating to the left arm, neck, jaw, which may be intermittent or persistent. Other symptoms associated may be diaphoresis, nausea, abdominal pain, dyspnoea, and syncope. However, atypical symptoms are not unusual [1]. A study has shown that between atypical presentation we might find in an ACS shortness of breath, nausea, diaphoresis, and pain or discomfort localized to other areas of the body such as the arm, epigastrium, shoulder, neck or jaw. In term of percentage it is shown that the most frequent symptoms associated with atypical presentation were dyspnoea (69.4%), nausea (37.7%), diaphoresis (25.2%), syncope (10.6%), or pain in the arms (11.5%), epigastrium (8.1%), shoulder (7.4%), or neck (5.9%) [2]. It does not discuss an atypical symptoms such as a low back pain, if not concerning the presentation and differential diagnosis of thoracic and abdominal aortic disease. Regarding this case report in the complex, this sintomatology, a low back pain, has been the spy of an ACS. A symptom such as this one is described associated with an ACS in other case report. Low-back pain has been presented during streptokinase infusion administered to treat typical chest pain and elevation of ST segment in the inferior wall [3]. In this interpretative key such a symptom is not so much the result of a full-blown ischemic disease, but rather of its treatment. In our case such a symptom precedes the onset, biochemical, and electrographic of the ischemic disease; on the contrary, in the preliminary steps of the development of what will be an ACS it is the first and only clue. In the study of atypical presentation of unstable angina, multivariate analysis showed that after adjusting for age, diabetes may be an important risk factor for atypical presentation in the elderly, but this did not meet statistical significance. Besides, prior studies have shown that diabetics during MI may not report pain, possibly due to an abnormality with the autonomic nervous system [4]. However, regarding the diabetes as a risk factor of the patient of this case and his history for abdominal aortic surgery, probably the patient to which we refer has had a disarray of his autonomic system and abnormality for mechanism of transmission of the visceral pain. In conclusion, it is possible that many clinicians may already recognize that the symptoms of dyspnoea, nausea, and diaphoresis in the absence of chest pain are typical of ischemia, and these results are like so this report should be used to heighten the awareness of patients at risk for atypical presentations, above all when clinicians take care of patients with previous history of heart disease, elderly, and diabetics.
